# Validation of a Dietary Questionnaire to Screen Omega-3 Fatty Acids Levels in Healthy Adults

**DOI:** 10.3390/nu11071470

**Published:** 2019-06-28

**Authors:** Wan Shen, Anne M. Weaver, Claudia Salazar, James M. Samet, David Diaz-Sanchez, Haiyan Tong

**Affiliations:** 1 Oak Ridge Institute of Science and Education, 100 ORAU Way, Oak Ridge, TN 37830, USA; 2 Department of Public and Allied Health, 119 Health and Human Services, Bowling Green State University, Bowling Green, OH 43403, USA; 3 Environmental Public Health Division, National Health and Environmental Effects Research Laboratory, U.S. Environmental Protection Agency, 104 Mason Farm Road, Chapel Hill, NC 27514, USA

**Keywords:** food frequency questionnaire, eicosapentaenoic acid, docosahexaenoic acid, omega-3 index, validation

## Abstract

To facilitate a clinical observational study to identify healthy volunteers with low (defined as ≤4%) and high (defined as ≥5.5%) omega-3 indices, a dietary questionnaire to rapidly assess habitual dietary intake of eicosapentaenoic acid (EPA) and docosahexaenoic acid (DHA) was developed. This study aimed to determine the validity of this newly developed dietary questionnaire. One hundred and eight volunteers were included and were assessed for habitual dietary intake of EPA and DHA using the questionnaire. The United States Department of Agriculture food products database and nutrition fact label was referenced for calculation. Blood samples were collected for the analysis of fatty acids in whole blood specimens and to derive omega-3 indices. A linear correlation was observed between reported dietary consumption of EPA, DHA, EPA+DHA and the whole blood levels of EPA, DHA, and the omega-3 indices (*r* = 0.67, 0.62, 0.67, respectively, *p* < 0.001 for all). The findings also suggested that the questionnaire was substantially better at identifying volunteers with high omega-3 indices (sensitivity 89%, specificity 84%, and agreement 86%) compared to volunteers with low omega-3 indices (sensitivity 100%, specificity 66%, and agreement 42%). In conclusion, this newly developed questionnaire is an efficient tool for the assessment of omega-3 indices in study populations and is particularly effective in identifying individuals with high omega-3 indices.

## 1. Introduction

Clinical trials are considered the gold-standard research approach in medical, biomedical, pharmaceutical, nutritional, and other health science-related fields. Identifying qualified volunteers is crucial for the success of the entire study, as the sample must closely represent the population of interest. However, volunteer eligibility screening often requires considerable labor and financial costs. The average direct contact time with the research staff and cost prior to the enrollment per study volunteer ranged from 3 to 9 h, and from $130 to $336, respectively [1], and in some disease- or stage-specific studies (such as diabetes and cancer-related studies), the prescreening or screening costs can be much higher [2,3,4]. Therefore, developing an effective and practical screening tool can be essential to the success of a clinical trial.

Omega-3 polyunsaturated fatty acids (PUFAs) are essential fatty acids. The newly published 2015–2020 Dietary Guidelines for Americans [5] recommends consumption of 8–10 oz/week of seafood to the general population to receive adequate amount of omega-3 PUFAs. Recently, omega-3 PUFAs have received great interest due to their potential benefits in cardiovascular [6,7,8,9], gastrointestinal [10,11,12,13], cognitive function [14,15,16], and bone health [17,18,19], as well as in pregnancy and offspring health outcomes [20,21,22,23]. The two omega-3 PUFAs associated with these biological effects are eicosapentaenoic acid (20:5, n-3; EPA) and docosahexaenoic acid (22:6, n-3; DHA). Alpha-linolenic acid (18:3, n-3; ALA), commonly found in nuts, seeds, and vegetable oils, is a substrate for the synthesis of EPA and DHA, however, the conversion is inefficient in the human body [24]. For example, only 0.2% of ALA is converted to EPA, and less than 1% ALA is converted to DHA in plasma [25]. Therefore, dietary intake of EPA and DHA is necessary for optimal health. The most common dietary sources for EPA and DHA include fatty fish (e.g., salmon, herring, mackerel), dietary supplements (e.g., fish oil, krill oil), and fortified food products (e.g., DHA fortified eggs). Positive correlations between the dietary consumption of EPA and DHA and their relative amounts in the blood lipid fractions have been documented [26].

The omega-3 index, which measures the levels of EPA and DHA in erythrocytes, was first reported in 2004 by Harris et al., and has been associated with an elevated individual risk factor for sudden cardiac death [27], coronary heart disease [28], depression [29], cognitive function [30], plasma levels of triglycerides and high density lipoprotein [31], attention deficit disorders [32], and non-alcoholic fatty liver disease in overweight and obese populations [33]. An omega-3 index of 8% or higher has been associated with a reduced risk of cardiovascular disease, while an index of 4% or lower is correlated with an increased risk of cardiovascular disease [27]. In the U.S., the average omega-3 index among healthy adult populations varied from 4.9 to 5.6% [34,35,36,37].

Studies have demonstrated that the omega-3 index is strongly correlated with plasma and whole blood level of EPA + DHA [26,27,38,39,40], as well as the frequency of dietary intake of seafood or fish oil supplements [38,39,40]. Sands et al. [34] recruited 163 healthy volunteers from the Kansas City Metropolitan area who were not taking fish oil supplements and found that volunteers with self-reported 1–2 servings/week (equals to 3–6 oz/week) consumption of tuna or nonfried fish had an average omega-3 index of 5.5%, whereas volunteers with self-reported less than 1 serving/mon (equals to 3 oz/mon) consumption of tuna or nonfried fish had an average omega-3 index of less than 4%. Therefore, whole blood levels of EPA and DHA, and omega-3 index were examined in this validation study and used as biomarkers for comparison with dietary intake of EPA and DHA. An omega-3 index of ≤4% or ≥5.5% was used for screening people with low versus high omega-3 PUFAs status in the body.

Dietary assessments, including questionnaires and dietary recalls, assess the diet quality and estimate individual nutrient consumption, and are commonly used as cost-effective research tools in healthcare practice and clinical research studies [41,42,43,44,45,46,47]. Although the food frequency questionnaire (FFQ) is easy to administer and able to capture the diet over an extended period of time, the majority of the validated FFQs (for example, FFQs developed by the National Cancer Institute, Block, or Harvard Willett) lack specificity [48], as they are usually applied to assess the overall quality of diet to a general population. Additionally, the amount of EPA and DHA from seafood, supplements, and fortified foods varies significantly. For example, one serving (3 oz) of farmed Atlantic salmon provides 0.587 g of EPA and 1.238 g of DHA (with a total of EPA + DHA 1.825 g), while one serving (3 oz) of cod provides only 0.003 g of EPA and 0.131 g of DHA (with a total of EPA + DHA 0.134 g) [49]. The standard FFQs mentioned above grouped multiple types of seafood together. Therefore, calculation of EPA and DHA is questionable in these FFQs. In fact, it is recommended that FFQs be designed to the specific focus of the research studies [48,50]. In the present study, we evaluate a brief and open-ended questionnaire specifically assessing frequency of habitual intake of different species of seafood, the United States Department of Agriculture (USDA) food products database is referenced when calculating for weekly intake of EPA and DHA.

This project, run in conjunction with an observational study, was aimed at determining the validity of a brief and open-ended dietary questionnaire as a screening tool for studies needing to identify volunteers with low (defined as 4% or lower in the study) versus high (defined as 5.5% or higher in the study) omega-3 indices.

## 2. Methods

### 2.1. Recruitment

This project was part of an ongoing observational study named PISCES (ClinicalTrials.gov registration number: NCT02921048) conducted at the U.S. Environmental Protection Agency Human Studies Facility at Chapel Hill, North Carolina. This study was designed to determine whether the high habitual (at least 6 months) dietary consumption of EPA and DHA (from seafood, fish oil or other omega-3 PUFAs supplements, and omega-3 PUFAs fortified packaged foods) protect healthy individuals against ambient air pollution induced cardiovascular and pulmonary dysfunctions. Healthy volunteers with low or high dietary consumption of EPA and DHA for at least 6 months preceding the study were targeted. A screening process was implemented in the study design, where the dietary questionnaire specifically assessing the habitual intake of dietary sources of EPA and DHA was designed. The validity of the dietary questionnaire used in the screening process is the focus of this manuscript.

This validation study was conducted from September 2016 to July 2018. The study protocol, recruitment materials, and consent forms were approved by the University of North Carolina at Chapel Hill Biomedical Institutional Review Board and the U.S. Environmental Protection Agency. All study volunteers were given informed consent and received monetary compensation for their participation.

**Figure 1:**
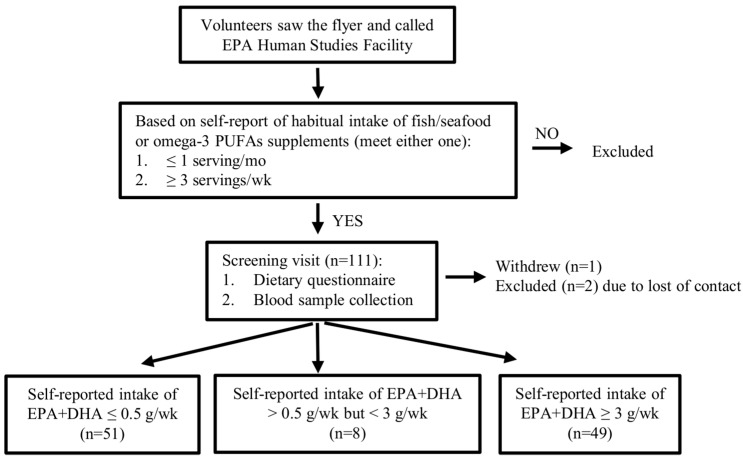
Study design. Based on the self-report of habitual intake of fish/seafood or omega-3 supplements, a total of 111 volunteers were invited for the screening visit where dietary questionnaire were conducted and blood samples were collected for fatty acids analysis. Nine volunteers were excluded from analysis due to the loss of contact or their dietary report did not meet the criteria.

Recruitment materials were distributed in the Research Triangle region and the surrounding area of North Carolina. Volunteers who were interested in the study were encouraged to call the recruitment service. During the first phone call, volunteers were asked for the general health condition, anthropometric and sociodemographic information including age, gender, race, marital status, education, self-reported height and weight. Frequency of intake of seafood or omega-3 supplements was specifically asked (Figure 1). Based on the answer, healthy volunteers aged 25 to 55 years-old (19 ≤ BMI ≤ 35) who self-reported with no more than 1 serving per month, or with no fewer than 3 servings per week (see “Determination of the dietary criteria” and “Omega-3 index and blood fatty acids levels”) of fish oil supplements or seafood in the past 6 months were invited for the screening visit. Then the dietary questionnaire (see “Dietary assessment questionnaire”) was administered to assess weekly consumption of EPA and DHA, while the blood samples were collected to measure whole blood fatty acids and omega-3 index (see “Omega-3 index and blood fatty acids levels”). Qualified volunteers were potentially invited to the observational study.

### 2.2. Determination of the Dietary Criteria

The Dietary Guidelines recommends general population take 8–10 oz/week of seafood, which provides an average of 0.25 to 0.5 mg/day (equals to 1.75 to 3.5 g/week) of EPA and DHA [51,52]. However, based on the NHANES 2003–2008 census, the average fish intake in the U.S. was less than 5 oz/week, of which less than 1.5 oz/week of fish containing high omega-3 fatty acids which was comparable to 0.25 to 0.5 g intake of EPA + DHA per week [53]. Therefore, for the recruitment of the observational study as well as this validation study, low omega-3 PUFAs intake was defined as ≤0.5 g/week of EPA + DHA, while the high omega-3 PUFAs intake was defined as ≥3 g/week of EPA + DHA.

### 2.3. Dietary Assessment Questionnaire

The in-house open-ended dietary questionnaire was designed to assess the habitual (more than 6 months) dietary consumption of EPA and DHA (Supplemental Figure S1). It contains four components. First, volunteers were asked whether they were following a special diet and how long they have been following it. They were also asked for all dietary supplements that they were currently taking. The second component asked their consumption of omega-3 PUFAs supplements. Information including brand names of the supplements, dose per serving, frequency, and duration for taking supplements was documented. The third section was designed to estimate volunteers’ habitual intake of seafood (taking on a regular basis). In this part, 29 items of seafood that are most commonly consumed near the U.S. east coast were listed, including bass, cod, halibut, herring, mackerel, salmon, sardine, tuna, trout, white fish, crab, shrimp, lobster, and mussel. Additional lines were used if reported items were not listed. Volunteers were asked to specify the kind of seafood in as much detail as possible. Cooking method, frequency, serving size and duration were also documented. In the last section, volunteers were asked about their habitual consumption of omega-3 PUFAs fortified foods such as omega-3 fortified eggs or milk. Types of omega-3 PUFAs fortified, frequency, serving size, and duration were recorded.

This paper questionnaire was designed and evaluated by a research-background registered dietitian. The administration of the questionnaire was conducted either by the registered dietitian or trained study personnel. Completion of the questionnaire may take 10 to 20 min. To help volunteers accurately estimate serving sizes, visual educational tools such as measuring cups were displayed and used consistently to verify the serving size. For the volunteers who took supplements or fortified foods, they were required to bring the products or email photos of the nutrition facts of the products so that the dose information can be correctly documented.

### 2.4. Calculation of Dietary Intake of EPA and DHA

The total consumption of EPA and DHA was calculated from their intakes of seafood, supplements, and fortified food products. USDA food products database [49] was used to calculate the consumption of EPA and DHA from fish, shellfish, and other seafood. The food product nutrition labels were referenced to calculate the consumption from the dietary supplements and fortified foods.

### 2.5. Omega-3 Index and Blood Fatty Acids Levels

Omega-3 index and whole blood levels of EPA and DHA were measured by OmegaQuant LLC. Blood samples were collected following the OmegaQuant kit instruction. Briefly, non-fasting blood samples were collected from a finger prick onto a filter paper, and the dry blood samples were then mailed to the company. Results of the omega-3 index and full blood fatty acids level were received via email from the company. The validity and reproducibility of measuring fatty acids and omega-3 index from the dry blood samples was previously published [54].

### 2.6. Sample Size

We hypothesized that the correlation between self-reported omega-3 PUFAs intake and blood levels of omega-3 PUFAs would be approximately 0.3 from the existing literature [55]. To detect correlations of 0.3, with 95% confidence and 80% power, we estimated a sufficient sample size to be 85. In this project, dietary and blood results from 108 volunteers were analyzed.

### 2.7. Statistical Analysis

Descriptive characteristics of volunteers, including age, gender, race/ethnicity, marital status, education level, and body mass index (BMI) were categorized based on self-reported levels of EPA and DHA intake: low (defined as habitual intake EPA + DHA ≤ 0.5 g/week), medium (defined as habitual intake of EPA + DHA > 0.5 g/week but <3 g/week), or high (defined as habitual intake of EPA + DHA ≥ 3 g/week). Data were expressed as Mean (Standard Deviation) for continuous variables or number (percentage) for categorical variables. Pairwise comparisons were made between the three groups (low versus medium, medium versus high, low versus high) using two sample *t*-tests for continuous variables and Fisher’s exact test for categorical variables.

To assess correlations between self-reported EPA, DHA, and EPA + DHA intake with blood levels of EPA, DHA, and omega-3 index, Pearson correlations were calculated between all indicators. Linear regression was conducted to estimate associations between self-reported EPA intake with plasma EPA, self-reported DHA intake with plasma DHA, and self-reported EPA + DHA intake with omega-3 index. Results were shown from crude models and models adjusted for all available potential covariates (age, gender, race/ethnicity, marital status, education level).

Two sample *t*-test was performed to detect group differences between volunteers who self-reported with the low intake, medium intake, or high intake of omega-3 PUFA. Sensitivity, specificity, positive predictive value, negative predictive value, agreement, and kappa were also reported based on the consumption level. Sensitivity was calculated as A/(A + B), where A = true positives (for example, classified as high intake from the questionnaire and high blood levels) and B = false negatives (for example, classified as not high intake from the questionnaire but high blood levels). Specificity was calculated as C/(D + C), where C = true negatives (for example, classified as not high intake and not high blood levels) and D = false positives (for example, classified as high intake but not high blood levels). Positive predictive value was calculated as (A + D)/A, and negative predictive value was calculated as (C+B)/C. Agreement was calculated as (A + C)/(A + B + C + D) × 100.

## 3. Results

### 3.1. Volunteer Characteristics and Blood Levels of Omega-3 PUFAs

A total of 111 healthy volunteers were enrolled for the screening visit for this validation study (Figure 1). One withdrew in the middle of the study. Two were excluded because of loss of contact. The main characteristics of volunteers were shown in Table 1. Out of the 108 volunteers who participated, 51 (47.2%) self-reported as taking low omega-3 PUFAs from the diet (the average consumption of EPA + DHA was 0.04 g/week calculated from the dietary questionnaire, low intake group); 49 volunteers (45.4%) self-reported as having a high weekly intake (the average consumption of EPA+DHA was 8.6 g/week, high intake group); eight volunteers (7.4%) self-reported having a medium intake of omega-3 PUFAs (the average consumption of EPA+DHA was 1.5 g/week, medium intake group). Distributions of age, gender, race/ethnicity, marital status, education, and BMI were similar between the low, medium, and high intake groups.

The average whole blood level of EPA, DHA, omega-3 index, and the omega-6:omega-3 ratio of the low, medium, and high intake group were 0.39, 0.54, and 1.2 for EPA; 2.2, 2.9, and 3.5% for DHA; 4.3, 5.2, and 6.6% for omega-3 index; and 9.6, 7.5, and 5.9 for the omega-6:omega-3 ratio (*p* < 0.05 for pairwise comparison among three groups and *p* < 0.001 when comparing between the low intake and high intake), respectively.

### 3.2. Dietary Consumption of EPA and DHA

Among the 108 volunteers, a total of 47 volunteers (43.5%) reported usage of omega-3 supplements including fish oil and krill oil across 24 commercial brands and store brands with an average of 1.2 servings per day (data not shown). Depending on the product, the content of EPA and DHA per serving varied from 0.01 to 2.53 g and 0.05 to 1.03 g, respectively. The lowest dosing was from an omega-3 gummy product, and the highest was from an omega-3 oil product.

Fifty-six volunteers (51.9%) reported consumption of at least one type of seafood regularly in the past 6 months. Among all seafood reported, salmon steak/fillet was the most frequently consumed, being regularly consumed by 43 volunteers with an average 1.9 servings per week (one serving equals to 3 oz or 85 g), followed by consumption of canned tuna (albacore or white) by 35 volunteers with an average 1.5 servings per week (Table 2). One serving of salmon fillet provides 0.34 to 0.59 g of EPA and 0.59 to 1.2 g of DHA depending on the origins and species and is thus widely considered as a good dietary source of EPA and DHA.

Regular consumption of EPA- or DHA- fortified food was not commonly reported (Table 2). Among the nine volunteers who reported any intake, the average number of servings consumed per week were 6.5 and 2.1 for DHA fortified eggs and DHA fortified milk, respectively. Generally, the DHA content in these fortified products was below 0.1 g per serving, as reported in the nutrition label.

### 3.3. Validation

**Figure 2:**
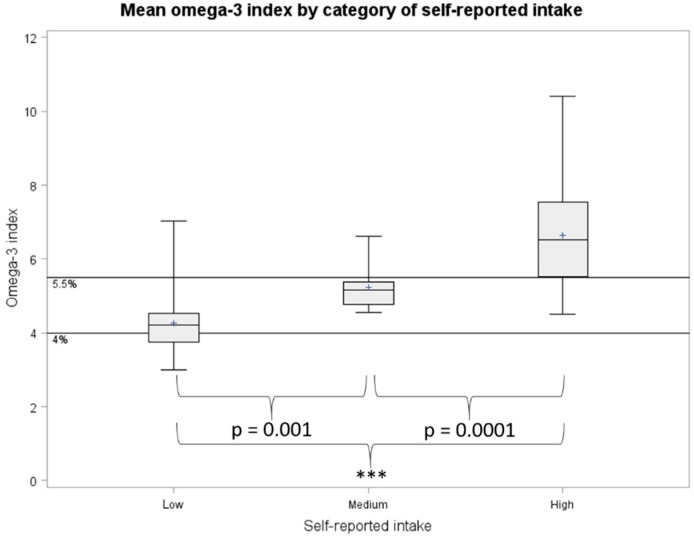
Boxplots of omega-3 index by self-reported intake of EPA + DHA. Low, self-reported habitual weekly intake of EPA + DHA ≤ 0.5 g; Medium, self-reported habitual weekly intake of EPA+DHA between 0.5 to 3 g; High, self-reported habitual weekly intake of EPA + DHA ≥ 3 g. *** *p* < 0.001 (two sample *t*-test).

The dietary questionnaire was designed to be used as a screening tool to identify volunteers with high and low omega-3 indices. In this study, the mean omega-3 index was 6.5% (SD 1.3%) from volunteers who self-reported high consumption of EPA + DHA (≥3 g/week) for the past 6 months, which was significantly higher than the omega-3 indices from the volunteers who reported low consumption of EPA + DHA (≤0.5 g/week) for the past 6 months (mean 4.3%, SD 0.76%, *p* < 0.0001, Figure 2). Volunteers with self-reported medium intake of EPA + DHA (0.5–3 g/week) had averaged omega-3 indices of 5.2% (SD 0.65%), which was significantly higher than the low intakers (*p* = 0.001) and lower than the high intakers (*p* = 0.0001).

**Figure 3:**
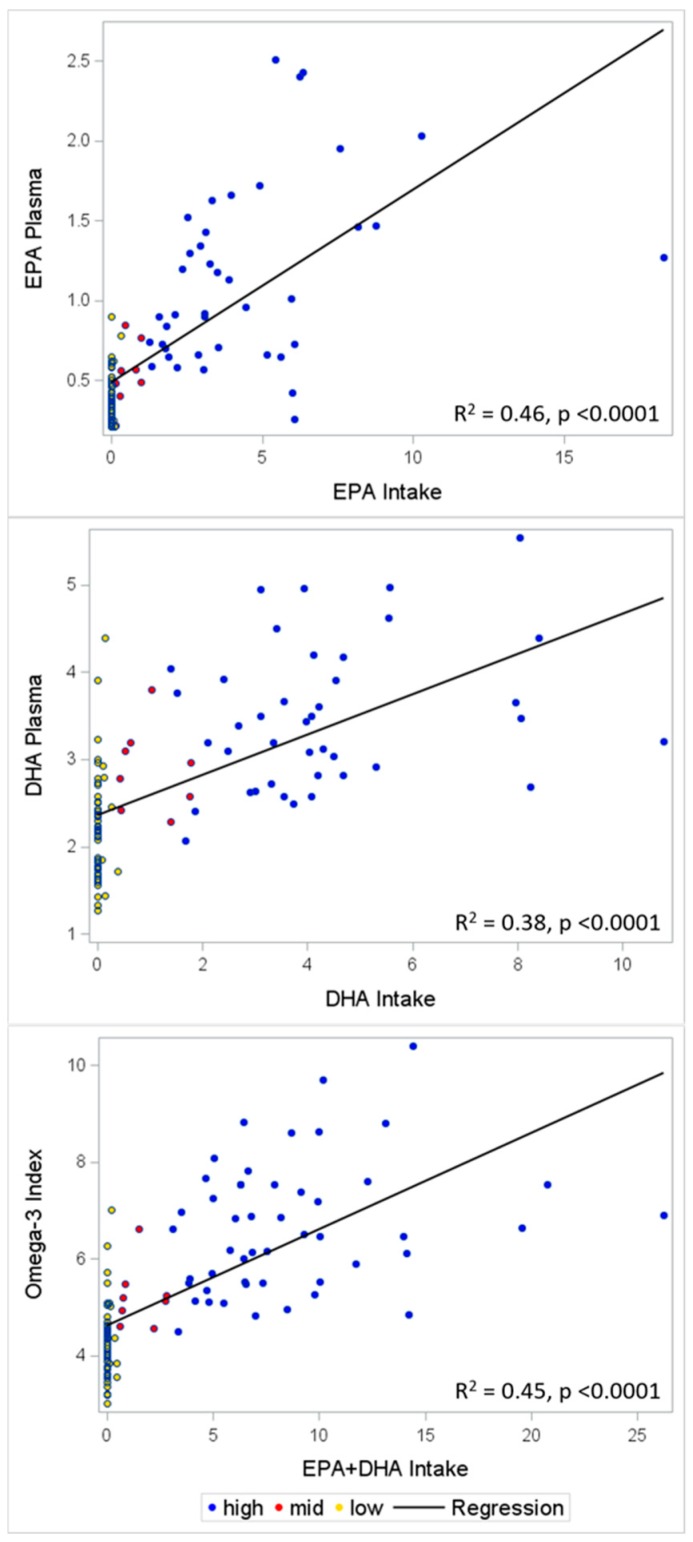
Scatterplots of blood levels of EPA, DHA, and omega-3 index by self-reported intake, respectively. Yellow dots were reported from volunteers with a self-reported habitual weekly intake of EPA + DHA ≤ 0.5 g, red dots were reported from volunteers with a self-reported habitual weekly intake of EPA + DHA between 0.5 to 3 g, blue dots were reported from volunteers with a self-reported habitual weekly intake of EPA + DHA ≥ 3 g. Regression lines were added in the figure.

The self-reported consumption of EPA, DHA, and EPA + DHA was highly correlated with whole blood levels of EPA (*r* = 0.67, *p* < 0.001), DHA (*r* = 0.62, *p* < 0.001), and omega-3 indices (*r* = 0.67, *p* < 0.001, Figure 3 and Table 3). Linear regression models indicated that, for each 1 g/week increase in intake of the specific omega-3 PUFAs, blood levels of EPA were 0.12% (95% CI 0.10–0.15%) higher, blood levels of DHA were 0.25% (95% CI 0.19–0.30%) higher, and omega-3 index was 0.20% (95% CI 0.16–0.24%) higher (Table 4) after adjusted for age, gender, race/ethnicity, education level, self-reported BMI, and marital status.

### 3.4. Sensitivity and Specificity Analyses

From sensitivity analyses, 100% of those with low omega-3 indices were correctly identified as having low intake from the questionnaire, and 89% (95% CI, 79–99%) of those with high-omega-3 indices were correctly identified as having high EPA + DHA consumption from the questionnaire (Table 5). From the specificity analysis, 66% (95% CI, 53–78%) of those who did not have low omega-3 indices were correctly identified as not having high EPA + DHA consumptions from the questionnaire, while 84% (95% CI, 75–94%) of those who did not have high omega-3 indices were currently identified as not having high EPA + DHA consumption from the questionnaire. Among the volunteers who reported low intake of EPA + DHA, 41% (95% CI, 32–50%) had omega-3 indices lower than 4% (positive predictive value), and of those who reported high intake of EPA + DHA, 80% (95% CI, 72–87%) of them had high omega-3 indices (positive predictive value). Among volunteers who did not report high EPA + DHA intake, 92% (95% CI, 86–97%) did not have the high omega-3 indices (negative predictive value). The agreement was 72% (95% CI 64–81%) for low intake and low omega-3 indices, and 86% (95% CI 80–93%) for the high intake and high omega-3 indices. Kappa was 0.42 (95% CI 0.28–0.57) for the low intake and low omega-3 indices and 0.72 (95% CI 0.59–0.85) for the high intake and high omega-3 indices, indicating that there is a fair chance that findings for low intake/low omega-3 index were not due to chance alone, and a good chance that findings for high intake/high omega-3 index were not due to chance alone.

## 4. Discussion

In seeking an efficient and practical screening tool to identify volunteers with high and low omega-3 indices, this dietary questionnaire was designed to assess individuals’ weekly dietary intake of EPA and DHA. The habitual weekly consumption of EPA and DHA calculated from the questionnaire was well-correlated with the blood level of EPA, DHA, and omega-3 indices (*r* = 0.67, 0.62, 0.67, respectively, *p* < 0.001 for all). In addition, there was good sensitivity, specificity, and agreement between the high intake of EPA+DHA and high omega-3 indices (sensitivity 89%, specificity 84%, and agreement 86%). The specificity and agreement between the low intake of EPA + DHA and low omega-3 indices was moderate (sensitivity 100%, specificity 66%, and agreement 42%).

The relatively lower numbers in specificity and agreement among volunteers with low omega-3 PUFAs intake and low omega-3 indices comparing with volunteers with high intake of omega-3 PUFAs and high omega-3 indices is possibly due to excluding dietary intake of ALA from calculation in this project. Although ALA is a substrate for production of EPA and DHA in the body, the conversation rate in healthy adults with regular diets is relatively low (0.2% for EPA and less than 0.1% for DHA) [24,56]. Therefore, the consumption of ALA from dietary sources, such as nuts (oil), seeds (oil), soy, and dairy products, was not designed to be recorded and calculated for analysis. However, it is possible that when exogenous EPA and DHA are limited, endogenous conversion from ALA becomes the dominant pathway to maintain the omega-3 PUFAs level in the body [57]. Rosell et al. [58] found that compared to meat-eaters, the plasma level of EPA and DHA in vegetarians (30% lower) and vegans (50% lower) are relatively low, but stable over time (≥16 years). Results from another cohort study by Welch et al. [59] suggested that the conversion efficiency from plant-derived ALA to plasma EPA and DHA might be significantly higher in non-fish eaters compared to volunteers who regularly eat fish. Therefore, when assessing individuals with low overall omega-3 PUFAs intake, dietary sources of ALA should be given consideration. In addition, excluding omega-3 PUFAs from diet may result in an imbalance of omega-6/omega-3 ratio, leading to modified bioactivities of delta-6 and delta-5 desaturases, which regulate the endogenous synthesis pathways of omega-3 and omega-6 PUFAs in the body [60]. Lastly, the questionnaire in this study assessed habitual intake of omega-3 PUFAs and occasional consumption of seafood or supplements were not counted into the analysis. It is likely that some volunteers had recently consumed seafood, but it was not reported as habitual intake.

To our knowledge, this dietary FFQ is the first designed to assess dietary consumption of EPA and DHA from specific species of seafood. The current FFQs [38,39,48] used for clinical studies especially the ones to study on omega-3 PUFAs grouped multiple types of seafood together and ask for frequency as a whole. However, due to the great difference in EPA and DHA content among species even within the fatty fish category, determination of dietary consumption of EPA and DHA is questionable and may affect research project results. In studies where traditional FFQs were used and frequency of grouped seafood were assessed, the correlation coefficient between the dietary omega-3 PUFAs intake and the blood lipid fractions has been reported between 0.21–0.39 [26]. In this study, the habitual dietary intake of EPA and DHA are calculated from seafood, supplement, and fortified foods, and therefore presented a higher correlation coefficient with blood levels of EPA (*r* = 0.67, *p* < 0.001), DHA (*r* = 0.62, *p* < 0.001), and omega-3 indices (*r* = 0.67, *p* < 0.001), respectively. In addition, a total of 8 volunteers were included in this validation study as medium omega-3 PUFAs intakers because they indicated they had at least three servings/week of seafood or/and omega-3 PUFAs supplements from the initial phone call with the recruitment staff but did not meet the study dietary criteria (EPA + DHA intake ≥ 3 g/week 6 months preceding the study). This observation indicates that using frequency of seafood or supplement intake may not accurately reflect actual dietary status of omega-3 PUFAs. Therefore, calculation of nutrients from specific dietary sources likely reflect more accurate dietary status and serve as a better screening tool for comparison to questionnaires that ask for frequency only, or those that group multiple foods together in a question.

The usage of krill oil supplements instead of the traditional fish oil or cod liver oil supplements has brought to our attention. In this study, two volunteers reported regular usage of krill oil supplements. Krill, a small crustacean from the Antarctic Ocean, is a rich dietary source of EPA and DHA and, therefore, a potential alternative to traditional fish oil or cod liver oil. The potency of polyunsaturated fatty acids contained in either krill meal or krill oil in increasing blood EPA and DHA has been reported [61,62,63], and has been found to be comparable or superior to the same dose of fish oil [61,64,65,66] due to the composition of fatty acids and their being attached to phosphatidylcholine instead of triglycerides in the fish oil. The dose per serving of krill oil reported in the study was 0.064 g of EPA and 0.03g of DHA, which were lower than a typical serving of fish oil supplements containing 0.18 g of EPA and 0.12 g of DHA (a total of 0.3 g of EPA and DHA) [51]. In this study, one volunteer reported an average of seven servings/week of krill oil and six servings/week of smoked salmon (containing 0.35 g/serving of EPA and 0.74 g/serving of DHA) in the past 9 months and had an omega-3 index of 5.5%, which was above the average omega-3 index of the U.S. population [34,35,36,37].

One limitation of this study is that volunteers with medium intake of EPA and DHA were not originally included due to the focus of the main observational study: to investigate the extent to which the habitual (at least 6 months) high dietary intake (≥3/week) of EPA + DHA protect healthy volunteers against ambient air pollution induced cardiovascular and pulmonary dysfunctions comparing to volunteers with low dietary intake (≤0.5 g/week) of EPA + DHA. Including data from the 8 volunteers whose intake of EPA and DHA falls in between 0.5–3 g/week (considered as medium intaker) may take the bias into this validation study as they do not present the population in this category: these 8 volunteers indicated they had at least 3 servings/week of seafood or/and omega-3 PUFAs supplements from the initial phone call but did not meet the study dietary intake criteria (EPA + DHA intake ≥ 3 g/week) based on the calculation from the developed dietary questionnaire. Future study is needed to address the validity of using this questionnaire to assess volunteers with medium intake of EPA and DHA (between 0.5–3 g/week). Two published FFQs that measure EPA and DHA intake were validated among healthy adults [67,68].

Additionally, this questionnaire was administered once to a single set of study participants. We were therefore unable to evaluate the reproducibility of these results. Future studies are needed to test the reliability of this questionnaire and potentially compare this questionnaire to other existing validated questionnaires.

Our current observational study is using the omega-3 index kit to screen potential qualified volunteers. The screening costs at least $200 per subject (including the cost for the blood analysis, reimbursement for the study screening visit and labor). The dietary questionnaire validated in this project is intended to assist screening process in clinical studies where levels of blood omega-3 fatty acids (particularly EPA and DHA) are of interest. The questionnaire can be conducted concurrent with other study visits (e.g., physical exam or training session) or potentially over the phone. A registered dietitian or a trained study personnel (e.g., research coordinator, nurse) can administer this screening.

From our experience, the administration of questionnaire can be completed within 20 min. During the visit, visual educational tools such as measuring cups and serving size tools are encouraged to use consistently to help volunteers accurately estimate their serving sizes. It takes 10 to 30 min to manually calculate the total intake levels of EPA and DHA: (1) calculate the intake levels of EPA and DHA from seafoods (reference the USDA online food products database https://ndb.nal.usda.gov/ndb/nutrients/index); (2) calculate the intake levels of EPA and DHA from supplements and fortified food products (reference product nutrition fact label), and (3) get the total intake of EPA and DHA by adding the corresponding numbers from step 1 and 2.

The questionnaire is free to use, and the USDA food products database is free online. Therefore, the cost of the screening is mainly from labor cost. In this project, the screening costs approximately $25 per subject ($50/h personnel cost), which is significantly lower than the cost for the blood test screening. In addition, results from the questionnaire screening can be obtained within 30 min, while the blood analyses of EPA, DHA and omega-3 index may take days. In the present project, the blood samples were mailed to the company and results were received via email after approximately 1–2 weeks.

## 5. Conclusions

In conclusion, the questionnaire developed in this study can be used as a free screening tool for studies in need of identifying volunteers with high (≥5.5%) or low (≤4%) omega-3 indices and is particularly effective in identifying individuals with high omega-3 indices. 

## Figures and Tables

**Table 1:** Subject characteristics and blood levels of omega-3 fatty acids ^1^.

	Low Intake (*n* = 51) ^2^	Medium Intake (*n* = 8) ^3^	High Intake (*n* = 49) ^4^
Age (years)	37.2 (8.8)	40.5 (8.3)	39.8 (9.9)
Gender			
Male	16 (31%)	1 (13%)	21 (43%)
Female	35 (69%)	7 (88%)	28 (57%)
Race/Ethnicity			
Non-Hispanic white	33 (65%)	3 (38%)	28 (57%)
African American	15 (29%)	4 (50%)	13 (26%)
Asian American	1 (2%)	0 (0%)	4 (8%)
Other ^5^	2 (4%)	1 (13%)	4 (8%)
Marital status			
Single	27 (53%)	5 (63%)	19 (39%)
Married	19 (37%)	3 (38%)	25 (51%)
Separated or divorced	5 (10%)	0 (0%)	5 (10%)
Education			
High school or trade school	6 (12%)	1 (13%)	6 (12%)
College	25 (49%)	1 (13%)	22 (45%)
Graduate school	20 (39%)	6 (75%)	21 (43%)
BMI (kg/m^2^) ^6^			
Self-report (*n* = 108)	24.7 (3.8)	23.7 (3.0)	25.3 (3.2)
Actual (*n* = 75)	25.8 (4.2)	22.6 (3.8)	25.6 (3.9)
Self-reported intake (g/week)			
EPA ^†^	0.013 (0.049)	0.52 (0.37)	4.5 (3.1) ***
DHA ^†^	0.025 (0.074)	1.0 (0.58)	4.3 (2.1) ***
EPA + DHA ^†^	0.038 (0.11)	1.5 (0.93)	8.6 (4.7) ***
Blood level (weight% from the whole fatty acids)			
EPA ^†^	0.39 (0.15)	0.54 (0.20)	1.2 (0.52) ***
DHA ^†^	2.2 (0.61)	2.9 (0.49)	3.5 (0.8) ***
Omega-3 index			
≤4% ^†^	21 (41%)	0 (0%)	0 ***
4–5.5% ^†^	26 (51%)	7 (88%)	10 (20%) ***
≥5.5% ^†^	4 (8%)	1 (13%)	39 (80%) ***
Omega-6:Omega-3 ^†^	9.6 (1.8)	7.5 (1.2)	5.9 (1.3) ***

^1^ Data were expressed as mean (standard deviation) or as number (percentage). ^2^ Self-reported intake of EPA and DHA ≤ 0.5 g/week. ^3^ Self-reported intake of EPA and DHA between 0.5 to 3 g/week. ^4^ Self-reported intake of EPA and DHA ≥ 3 g/week. ^5^ Other race include white Hispanic and other races. ^6^ BMI, body mass index. Self-reported BMI was calculated from self-reported height and weight. All volunteers self-reported height and weight during the first phone contact. Actual BMI was calculated from measured height and weight. Measured height and weight was obtained from the primary observational study or U.S. EPA medical station volunteer database (updated annually). Thirty-one volunteers from the Low intake group and 40 volunteers form the high intake group were measured for actual height and weight. ^†^ significantly different within all groups using pairwise comparisons, *p* < 0.05 *** significantly different from the “Low intake” group using student *t* test, *p* < 0.001.

**Table 2:** Frequency of consumption of seafood and omega-3 fatty acids fortified food items.

Food Item	Number of People Reported any Intake	Averaged Servings Consumed per Week (Servings) ^1^		Averaged EPA Content per Serving ^2^	Averaged DHA Content per Serving ^2^
		Mean	Range	(g/Serving)	(g/Serving)
**Seafood**					
Bass, seabass	4	1.69	0.75–3	0.175	0.473
Catfish, farmed	1	1	na ^3^	0.042	0.109
Clams	2	0.32	0.3–0.33	0.117	0.124
Cod	7	1.3	0.33–3	0.003	0.131
Crab	7	2.88	0.33–16	0.251	0.1
Flounder	5	1.22	0.5–3	0.207	0.219
Halibut	1	0.25	na	0.077	0.318
Herring, canned	2	0.75	0.5–1	0.825	1.002
Grouper	1	0.5	na	0.03	0.181
Lobster	2	0.63	0.25–1	0.29	0.118
Mackerel, canned	4	3.08	1.33–6	0.369	0.677
Mahi Mahi	1	1	na	0.022	0.096
Oyster, farmed	2	0.5	0.5–0.5	0.195	0.179
Porgy	1	0.25	na	0.088	0.451
Salmon ^4^					
Canned	3	1.21	0.67–1.67	0.402	0.597
Steak	43	1.92	0.25–6.67	0.341–0.587	0.595–1.238
Sardine, canned	5	1.02	0.67–2	0.402	0.433
Scallop	1	1.33	na	0.141	0.169
Shrimp	17	1.73	0.25–5	0.145	0.122
Snapper	1	1	na	0.041	0.232
Tilapia	11	1.62	0.5–4	0.004	0.111
Trout, rainbow, farmed	6	0.7	0.25–2	0.284	0.697
Tuna ^4^					
Canned, albacore or white	35	1.54	0.25–8.33	0.198	0.535
Steak	6	0.74	0.33–1.33	0.04–0.309	0.19–0.97
**Fortified food**					
DHA fortified eggs ^5^	5	6.5	1.5–12	0	0.075
DHA fortified milk	4	2.1	1–3.5	0	0.032

^1^ For calculation purposes, one serving equals to 3 oz or 85 g. ^2^ EPA, eicosapentaenoic acid; DHA, docosahexaenoic acid. EPA and DHA content from each serving were referenced from the United States Department of Agriculture food products database. Values were based on dry heat cooking methods unless otherwise mentioned. ^3^ na: not applicable. Ranged not applicable due to only one person reported consumption. ^4^ EPA and DHA content varies based on the species and origins of the fish. ^5^ DHA content was obtained from the product nutrition fact label.

**Table 3:** Person correlation matrix between self-reported intake of EPA and DHA, and blood levels of EPA, DHA and omega-3 index.

	Self-Reported EPA Intake	Self-Reported DHA Intake	Self-Reported EPA + DHA Intake	EPA Level in the Blood	DHA Level in the Blood	Omega-3 Index
Self-reported EPA intake	1					
Self-reported DHA intake	0.82 ***	1				
Self-reported EPA + DHA intake	0.96 ***	0.95 ***	1			
EPA level in the blood	0.67 ***	0.67 ***	0.68 ***	1		
DHA level in the blood	0.51 ***	0.62 ***	0.59 ***	0.71 ***	1	
Omega-3 index	0.62 ***	0.69 ***	0.67 ***	0.87 ***	0.96 ***	1

*** Correlation coefficient (*r*) statistically significant, *p* < 0.001.

**Table 4:** Linear regression of self-reported intake of EPA, DHA, and EPA+DHA on blood levels of EPA, DHA and omega-3 index, respectively.

	EPA	DHA	Omega-3 Index
	β (95% CI)	β (95% CI)	β (95% CI)
Self-reported intake			
Crude	0.12 (0.09, 0.15)	0.23 (0.17, 0.29)	0.20 (0.16, 0.24)
Adjusted ^1^	0.12 (0.10, 0.15)	0.25 (0.19, 0.30)	0.20 (0.16, 0.24)

^1^ adjusted for age, gender, race/ethnicity, education level, BMI (based on self-reported height and weight), and marital status.

**Table 5:** Sensitivity, specificity, positive predictive value, and negative predictive value based on the category: self-reported low (≤0.5 g/week) and high (≥3 g/week) EPA + DHA intake.

Reported Intake	Sensitivity	Specificity	Positive Predictive Value	Negative Predictive Value	Agreement	Kappa
	(95% CI)	(95% CI)	(95% CI)	(95% CI)	(95% CI)	(95% CI)
Low	100% (100%, 100%)	66% (53%, 78%)	41% (32%, 50%)	100% (100%, 100%)	72% (64%, 81%)	0.42 (0.28, 0.57)
High	89% (79%, 99%)	84% (75%, 94%)	80% (72%, 87%)	92% (86%, 97%)	86% (80%, 93%)	0.72 (0.59, 0.85)

## Supplementary Materials

The following are available online at https://www.mdpi.com/2072-6643/11/7/1470/s1, Figure S1: Dietary questionnaire.

## Abbreviations

ALA	alpha-linolenic acid
BMI	body mass index
DHA	docosahexaenoic acid
EPA	eicosapentaenoic acid
FFQ	food frequency questionnaire
PUFAs	polyunsaturated fatty acids
USDA	United States Department of Agriculture
