# Supplementary material for: Validation of a Dietary Questionnaire to Screen Omega-3 Fatty Acids Levels in Healthy Adults
Source: Nutrients. 2019 Jun 28;11(7):1470. doi: 10.3390/nu11071470 (PMC6682879; doi:10.3390/nu11071470)
Supplement: Supplementary file 1 [file nutrients-11-01470-s001.pdf]

Supplemental Figure 1. Dietary questionnaire

Preliminary Dietary Assessment

(Focusing on consumption of DHA and EPA)

a. Do you currently follow a special diet?

b. How long have you been following the diet you mentioned above?

c. Do you currently take any dietary supplements? Please list them all and the frequency.

Section 1. Consumption of fish oil supplements.

Question 1. Fish oil supplements (pills)

- a. Which kind (including brand)? ______________________________
- b. Dose of each pill? ________________________________
- c. How often do you take the pills (how many times per week)? ________
- d. How many pills each time? ______________________________________
- e. How long have you been taking fish oil supplements? ___________

Question 2. How often do you take fish oil (liquid)?

- a. Which kind (including brand)? _________________________
- b. Dose per serving (from the label)? ________________________
- c. How often do you take oil (how many times per week?)? ______________________
- d. How much do you take fish oil each time? ____
- e. How long have you been taking the supplements? _______________

Comments:

Section 2. Consumption of fish and shellfish.

Question 1. Have you been taking any of the following items on a WEEKLY basis in the past 6 month? Please include all ocean fish/shellfish that you eat regularly (at least on a weekly basis) in the past 6 month in this section. Use your best estimation for serving size.

Example: Tuna, canned, 3 times a week, 2 tablespoon.

Food item	Kind	Cooking method	Frequency	Serving size	How long
Anchovy					
Bass					
Bluefish					
Cisco					
Cod					
Drum					
Halibut					
Herring					
Mackerel					

Food item	Kind	Cooking method	Frequency	Serving size	How long
Pollock					
Sablefish					
Salmon					
Sardine					
Shark					
Shad					
Smelt					
Swordfish					
Tuna					
Trout					
Whitefish					
Wolfish					
Caviar					

Food item	Kind	Cooking method	Frequency	Serving size	How long
Roe					
Crab					
Lobster					
Mussel					
Oyster					
Shrimp					
Squid					

Section 3: Consumption of omega-3 fortified foods.

Question 1: Have you been taking any omega-3 fortified foods on a WEEKLY basis in the past 6 months? Please include them all in this section.

Food item	type of ω-3 FA	Frequency	Serving size	How long

Comments:
